# Interacting with virtual objects via embodied avatar hands reduces pain intensity and diverts attention

**DOI:** 10.1038/s41598-021-89526-4

**Published:** 2021-05-21

**Authors:** Hunter G. Hoffman

**Affiliations:** 1 The Virtual Reality Analgesia Research Center at the Human Photonics Lab, University of Washington, Box 352142, Seattle, WA USA; 2 Computer Science, King Abdulaziz University, Jeddah, Saudi Arabia

**Keywords:** Psychology, Human behaviour

## Abstract

The current study introduces a new paradigm for exploring cognitive factors in pain. Interacting with virtual objects via embodied avatar hands increased the illusion of “being there” in the virtual world, increased VR analgesia for acute pain, and reduced accuracy on an attention demanding task. Twenty-four healthy volunteer college students participated in this within-subject randomized crossover design study. During Phase 1, each participant received brief thermal pain stimuli during interactive embodied avatar VR vs. passive VR (no avatar and no interactivity), VR treatment order randomized. After each pain stimulus, participants provided subjective 0–10 ratings of pain. Compared to the passive VR condition, during the interactive avatar VR, participants reported significant reductions in (1) worst pain, (2) pain unpleasantness, (3) time thinking about pain and (4). they had significantly more fun during the pain stimulus (*p* = .000 for each). During Phase 2, participants performed a divided attention task in each of the two VR conditions. Participants made significantly more errors on the divided attention task during the interactive avatar VR condition, compared to passive VR, implicating an attention mechanism for how virtual reality reduces pain and helping understand how VR influences pain perception.

Trial registration: NCT04245475. Date of registration: 29/01/2020.

## Introduction

The opioid crisis makes development of non-opioid pain control techniques a national priority^1^. Pain levels during medical procedures are frequently excessive^2,3^. Opioid analgesics help reduce pain, and become more effective at higher doses^4–6^, but opioid side effects limit dose levels^7^. Furthermore, unhelpful psychological influences can exacerbate/increase pain intensity during wound care^8–12^, and excessive acute pain can lead to chronic pain^13^. Fortunately, adjunctive psychological treatments can help reduce pain, with few or no additional side effects. For example, immersive virtual reality, first reported by our team in the 1990s^14–16^, is emerging as an unusually powerful adjunctive non-pharmacologic analgesic^1,17–26^. fMRI brain scans show that in addition to reducing participants’ subjective experience of pain, VR also reduces pain-related brain activity^27,28^. A follow up fMRI study showed that VR reduces pain as much as a moderate dose of hydromorphone^28^. However, the mechanism of how VR reduces pain and why some VR systems are more effective than others, is not well understood and is the focus of the current study.

**Figure 1:**
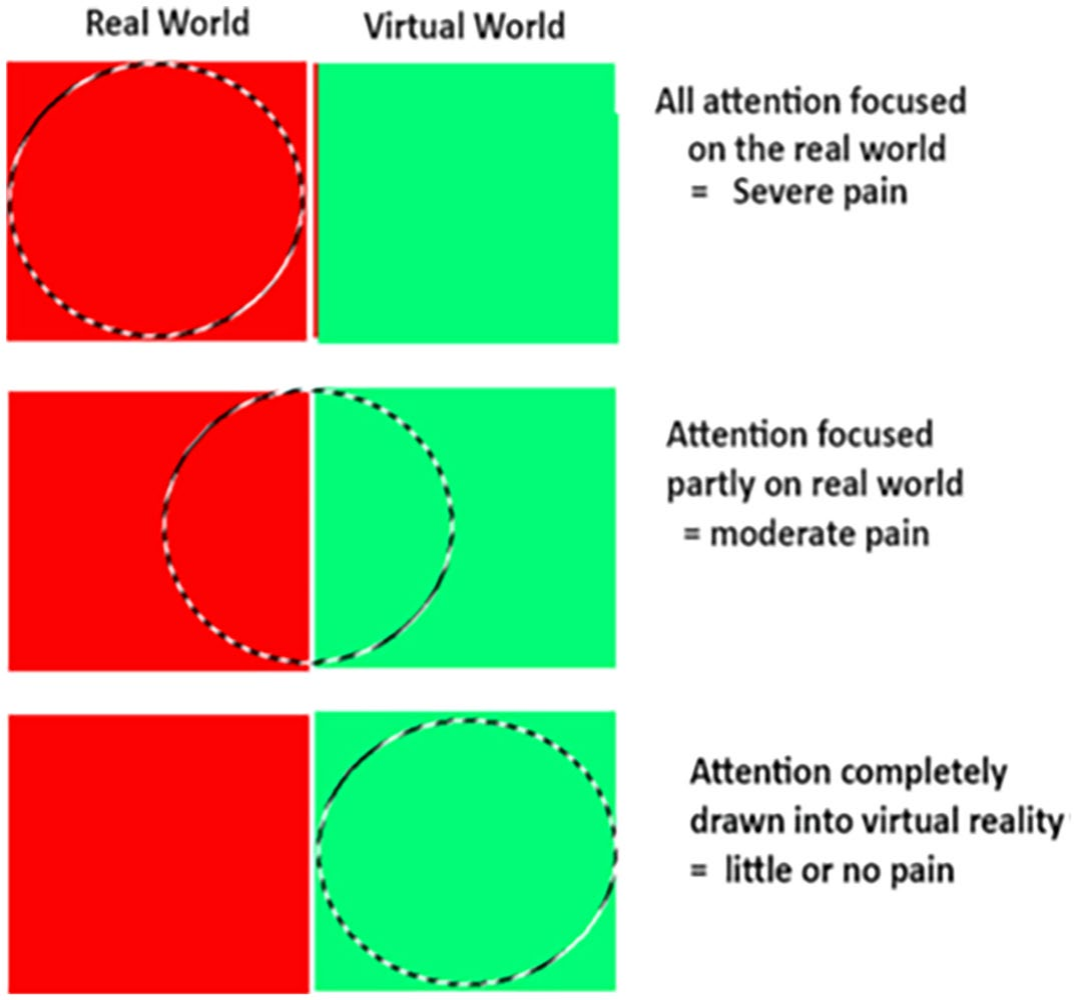
Increasing the illusion of “being there” in VR may increase the amount of attention drawn into VR, leaving less attention available to process incoming nociceptive signals. If so, participants may feel less pain during Avatar VR. Copyright Hunter Hoffman, www.vrpain.com.

Considering VR Analgesia to be a divided attention task may help understand how VR reduces pain and why some VR systems reduce pain more effectively than other VR systems. During VR analgesia, instead of devoting all of their attention to pain, during VR, some of the patient’s attention is used to process the information coming into their brain from the VR system, and simultaneously, some of their attention is used to process information coming into their brain from the pain receptors (e.g., in their skin). In other words, during VR, some of the brains attentional resources are allocated to VR, and some attentional resources are allocated to pain. The more attention grabbing the VR system, the less remaining attention the patient’s brains have available to process incoming nociceptive signals, and the more effective the VR analgesia treatment (see Fig. 1).

In the current context, embodiment (e.g., “in a body”) involves giving participants a sense of ownership over a computer generated representation of the participant, aka, an avatar^29^, for example “these cyberhands I see in VR, are my hands.” In the current study, I predicted that interacting with virtual objects via embodied avatar hands would increase the subject’s illusion of “being there” in the virtual world, would make VR more attention grabbing, would increase VR analgesia, and would reduce accuracy on an attention demanding task, implicating an attentional mechanism for how VR reduces pain.

## Methods

### Participants and recruitment

Twenty-four college students from the University of Washington Department of Psychology (18–24 years old, mean = 18.93 years old, SD = 1.36) were included in the main study, and an additional different 12 students participated in a side project to test the assumptions of our pain paradigm (age range 18–24). Participants in the side study received passive VR for both test phase thermal pain stimuli (instead of one passive VR and one interactive cyberhands VR condition used in the main study). All data was collected between late January and early March 2020. All participants included in each analysis were in their originally assigned treatment order groups.

*Inclusion criteria:*

- Currently enrolled in a course at the University of Washington Psychology Dept., participating in the UW Psychology subject pool
- Able to read, write and comprehend English
- Able to complete study measures
- Willing to follow our UW approved instructions
- 18 years of age or older

*Exclusion criteria:*

- Not be able to read, write and comprehend English
- Younger than 18 years of age
- Not capable of completing measures
- Extreme susceptibility to motion sickness
- Seizure history
- Unusual sensitivity or lack of sensitivity to pain
- Sensitive skin
- sensitive feet
- Migraines
- Diabetes.

This research was conducted in accordance with the Declaration of the World Medical Association. All participants gave written informed consent in accordance with the Declaration of Helsinki. Both written and verbal informed consent were obtained using a protocol approved by the University of Washington Human Subjects Review Committee. Informed consent was obtained for publication of any identifying information/images in an online open-access publication.

ClinicalTrials.gov Identifier: NCT04245475, date of registration 29/01/2020.

### Experimental design: Within-subjects design

#### Phase 1: thermal pain stimulation

**Figure 2:**
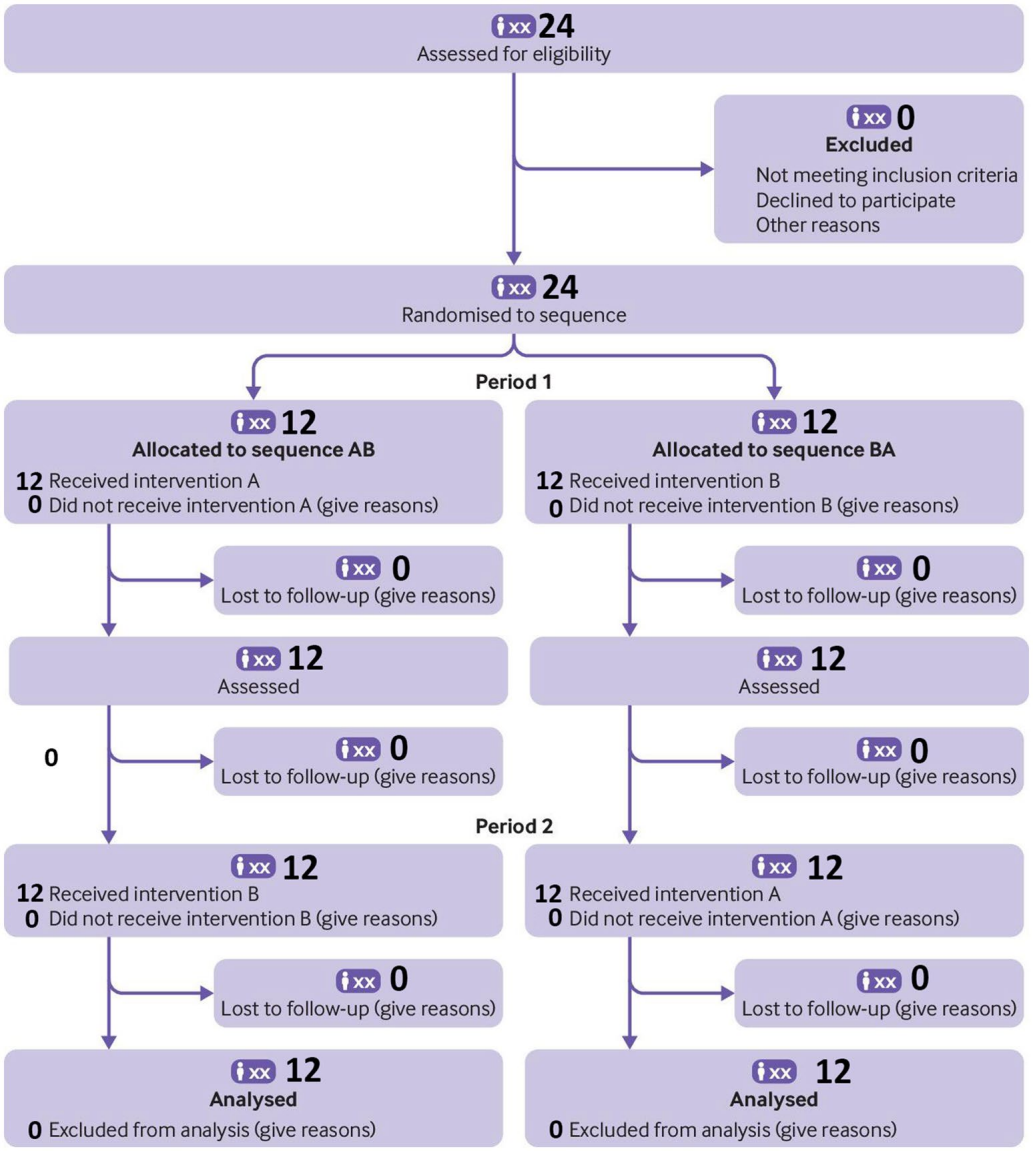
Participants’ enrollment flowchart.

**Figure 3:**
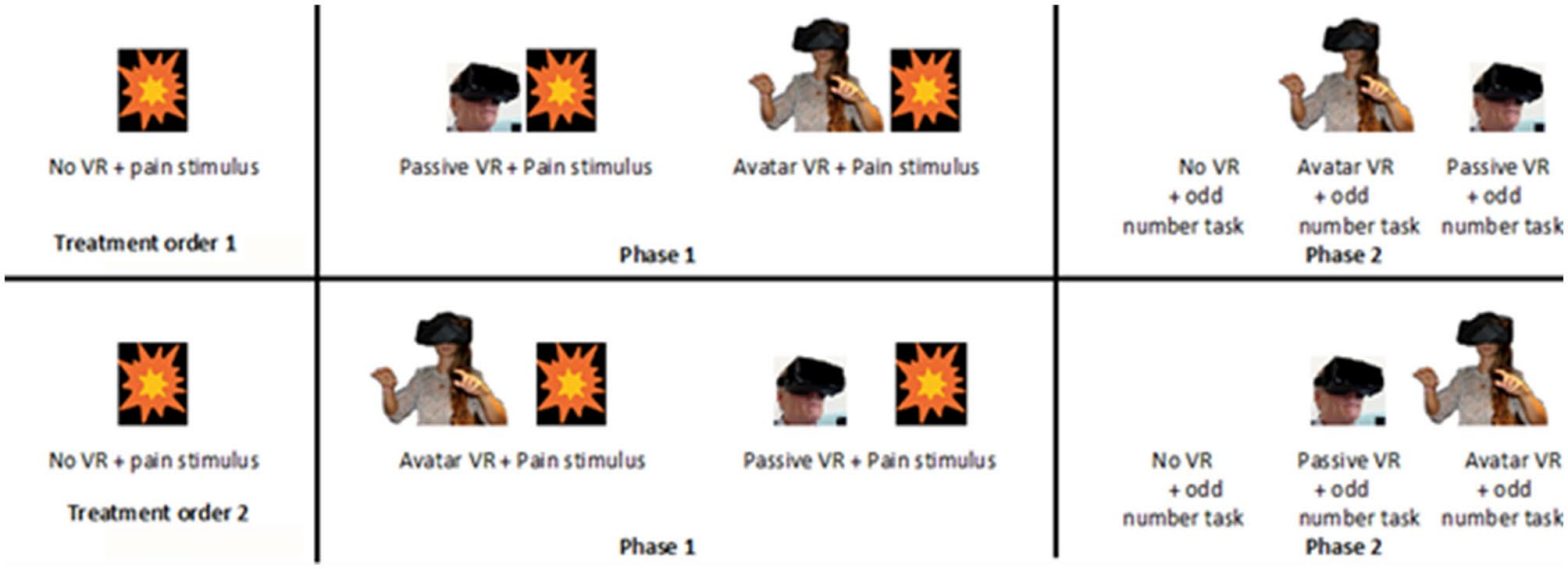
A detailed description of the stimulus sequence. Participants are randomly assigned to either Treatment Order 1 or Treatment Order 2.

In addition to a baseline “no VR” thermal pain stimulus and pain rating, each of the 24 participants in the main study rated their pain during “passive VR with no cyber hands and no interactivity” during one thermal pain stimulus, and after a brief inter stimulus interval wash out period, rated their pain again during “yes interactive cyberhands avatar VR” during a second thermal stimulus. To help balance the number of participants in each treatment order, VR treatment order was block randomized using random number sequences from www.random.org. The random numbers were written on 2” paper squares, and each paper square was put into its own sealed sequentially numbered opaque envelope and was opened by the researcher as the subject was reading the consent form. To reduce bias, the researcher only learned the treatment order of an individual participant during the consent process. Participants knew they would receive two VR treatments but they only learned the details of each VR treatment (passive vs. interactive cyberhands) during the brief instructions immediately preceding each VR treatment. Using an AB/BA within subject design, with treatment order randomized, some participants received “interactive cyberhands VR” first and “passive VR with no cyberhands” second, and others received “passive VR with no cyberhands” first and “interactive cyberhands VR” second, (see participant enrollment flowchart in Fig. 2). “The particular strength of the simple AB/BA crossover design is that both interventions are evaluated using the same participant, which allows comparison at the individual rather than the group level” (Dwan et al. 2019, page 2)^30^. The within-subject design reduces nuisance variance. The VR analgesia + odd numbers protocol described herein was developed to measure for the first time how effectively a given virtual reality system reduces acute pain, and the relation between the amount attention allocated to VR, and VR analgesia effectiveness of that VR system. The current experiment involves the identification of a painful but tolerable temperature (baseline pain, no VR). After the baseline pain temperature is identified, Phase 1 begins. Phase 1 measures pain during VR. Participants experience a High Tech VR treatment during one brief pain stimulus, and they experience a Low Tech VR condition during another brief stimulus (VR treatment order randomized). Phase 2 (attention during VR) measures performance on a brief cognitive test designed to quantify how much attention is paid to the test during No VR, during High Tech VR and during Low Tech VR, by measuring the participants accuracy on the attention demanding “odd number” task during No VR, during Low Tech VR, and during High Tech VR (See Fig. 3).

#### Phase 1: quantitative sensory testing

Following the paradigm used by our team in several previous studies^27,28^, a commercially available Medoc Quantitative Sensory Testing thermal (heat) pain stimulator (Ramp and Hold program) was used to deliver brief 10 s heat stimuli to their right foot, at “painful but tolerable” temperatures individually pre-selected by each participant. During Phase 1, each student was allowed to choose the temperature they wanted to use during the test phase. The mean stimulus temperature selected by participants was 45.2 °C (SD = 1.40, range = 44–48.0 °C).

Participants received the following instructions. “During Phase 1, you will receive several 10 s pain stimuli. Starting at a relatively low temperature, we will slowly work our way up, one 10 s stimulus at a time, to a temperature you find painful but tolerable. You get to select which temperature to use and once you select the temperature you want to use during the test phase, you will receive only two additional 10 s stimuli at one of the temperatures you pre-approved. Phase 1 typically lasts around 10–15 min. We are not trying to see the highest pain you can tolerate, we are just wanting you to find a temperature that you find ‘painful but tolerable for 10 s’, that you are willing to experience for two additional 10 s stimuli, later in the study.”

The instructions to participants for GRS ratings were as follows: “Please indicate how you felt during the most recent 10-s pain stimulus by making a mark anywhere on the line. Your response does not have to be a whole number.” The above two sentences of instructions were presented to the subject on a conventional laptop computer screen, using a computer animated text-to-speech character software program https://ttsdemo.com/. The researcher previously typed the above two sentences of instructions into the text to speech website, and when the researcher clicked start, the animated character on the laptop screen said the brief instructions to the participant. The animated text to speech character was used to make the animated “research assistant” blind to treatment condition for at least part of the study, to help reduce bias.

After each brief thermal stimulus, participants indicated how painful they found the stimulus using Graphic Rating Scales (GRS), validated by the measures’ strong associations with other measures of pain intensity, as well as through the measure’s ability to detect treatment effects^31–33^. GRS ratings were used to measure “worst pain”, “pain unpleasantness”, and “time spent thinking about pain” that correspond to three separable components of the pain experience; sensory pain, affective pain, and cognitive pain, respectively.

The question regarding “to what extent did you feel like you ‘went into’ the virtual world,” was adapted from Slater et al.^34–36^ Similar presence measures have been shown to be reliable and able to detect treatment effects^37–41^.

### Equipment

The VR laptop computer MSI GeForce GTX 1080 8 GB, Intel Core i7 7th (2.80 GHz), 16 GB RAM, Windows 10 operating system was connected to a VRGineering.com XTAL VR helmet, 5120 × 1440 (2560 × 1440 per eye), with extra wide 170–180 degrees field of view, using Two Quad HD high-density OLED displays.

A VR demo Architecture World (vrgineering.com) that came with the helmet, included optical hand tracking of participants’ real hands to simultaneously control their cyberhands in VR. The XTAL VR helmet came with LEAP Motion camera based optical hand tracking https://www.leapmotion.com/. The VR system was designed to give participants the illusion of “being there” in a 3D computer generated virtual house named Architecture World (VRgineering.com).

In both VR treatment conditions, each subject could see a living room in virtual reality, with a green living room chair, and two books sitting on a coffee table next to the green chair. Architecture World had no audio or sound effects in the current study.

### Phase 1: measuring pain during passive VR vs. during interactive cyberhands avatar VR

For the passive VR condition, participants received the following instructions. “*For this next part of the study, please put on the VR helmet, you will see a living room, and will look at a green chair, and a coffee table with books on it. While you are in VR doing this, you will receive another 10 s pain stimulus at one of the temperatures you have approved. In other words, we won’t go any higher than that last stimulus you approved*”. After receiving a brief thermal stimulus while in virtual reality, participants took off the VR helmet and answered the pain ratings, using the pen and paper GRS pain and presence ratings. The pain ratings and subsequent instructions phase lasted approximately 5 min, and served as the “wash out” period between VR treatments, to help minimize carryover effects.

Participants received the following instructions for the Phase 1 interactive embodied cyberhand avatar VR analgesia condition. “*For this next part of the study, please put on the VR helmet, you will see a living room, and will look at a green chair, and a coffee table with books on it. Please reach out with your hand and grab one of the virtual books, drop it on the floor, grab the second virtual book, drop it on the floor and then wiggle your fingers the rest of the time. While you are in VR doing this, you will receive another 10 s pain stimulus at one of the temperatures you have approved. In other words, we won’t go any higher than that last stimulus you approved.*”

**Figure 4:**
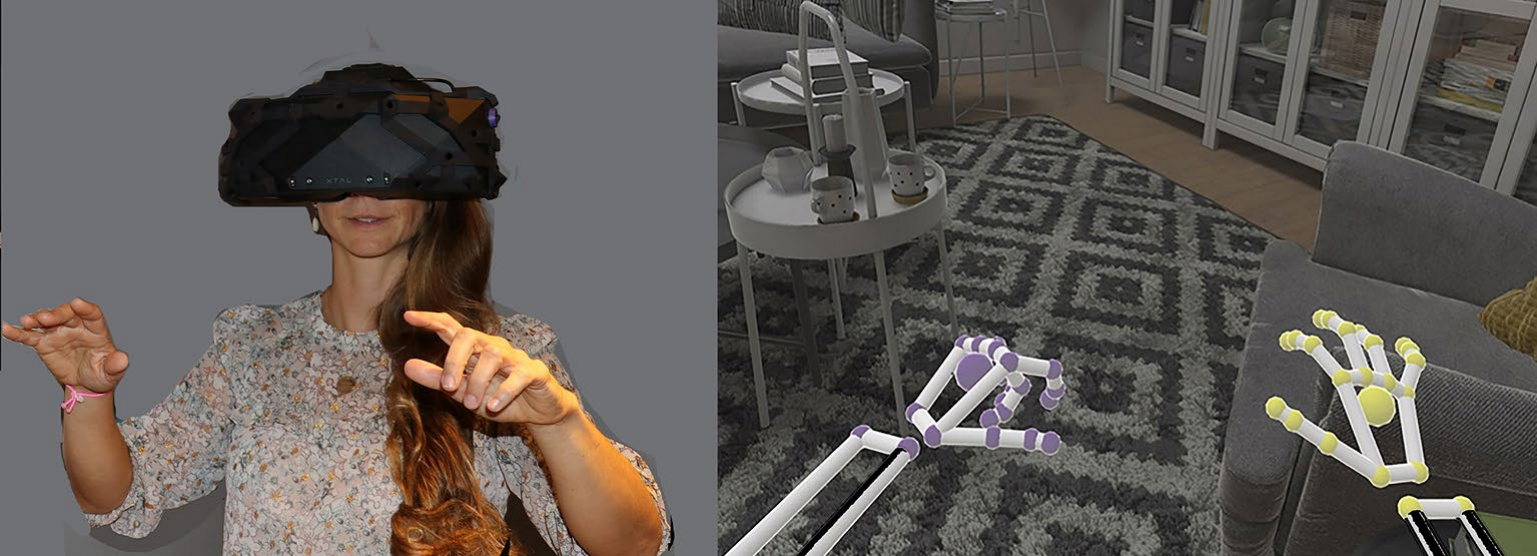
While wearing the XTAL VR helmet, any movements of the hands or fingers in the real world are seen by the participant in the virtual world (the image on the right is from the Architecture World demo by VRgineering.com). Photo and image copyrights Hunter Hoffman, U.W., vrpain.com.

While in virtual reality, during the interactive embodied cyberhands avatar condition, participants could interact with objects in virtual reality using their hand movements in the real world (see Fig. 4). For example, they could wiggle the fingers of their cyberhand in VR by wiggling their real fingers whose detailed motion was detected by a miniature head mounted video camera attached to the XTAL VR helmet (the video camera facing outward towards the real world). With the camera based LEAP Motion system, patients did not have to hold any controllers or hardware in their hands, and did not have to wear cybergloves. The patient wearing virtual reality goggles could see their virtual hands in the virtual living room, and could use their cyberhands to interact with virtual objects in the computer generated world (pick up virtual books and drop the books onto the virtual living room floor, see Fig. 4). All participants were able to perform all tasks. Afterwards, they took off the VR helmet and answered the pain ratings, using the pen and paper GRS pain and presence ratings.

### Phase 2: measuring the amount of attention used by passive VR with no avatar vs. interactive avatar cyberhands VR

After Phase 1, the thermal pain stimulator was removed from the participants foot, and participants were told that Phase 1 was now over, and they received the following instructions. “You have just completed Phase 1 and you can now remove the thermal pain stimulator from your foot. Please take off the thermal stimulator and put your shoe back on now. There are no more pain stimuli, but we will now begin Phase 2 of the study. In Phase 2, no pain stimuli will be administered. Instead you will go into virtual reality again and while you are in the virtual world, you will monitor a string of numbers from 1 to 10, and will say ‘now’ any time you hear three odd numbers in a row, (the odd numbers are 1, 3, 5, 7 or 9). For example, if you hear “1, 9, 3”, you would say “now”. The researcher will keep track of your answers and will be measuring your accuracy on the odd number task. You will receive a total of three divided attention tasks which each last 2 min per task. The first two minute session is with No VR. Are you ready to begin the odd number task? Just say “now” any time you hear 3 odd numbers in a row.

The researcher then played the pre-recorded auditory string of numbers (see below) via a digital audio file on a laptop with high quality Dolby Sound laptop speakers. The traditional odd number divided attention task^42–45^ was adapted for use in the current VR study, using our own new number set (shown below) customized for the present study (with 10 odd number triads during a 2 min session).
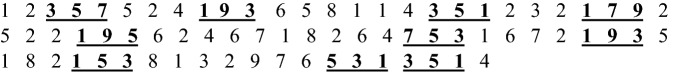


The string of numbers (i.e., the odd number task) lasted 2 min. The same identical two minute audio file was played a total of three times. The first time, it was played with No VR (baseline). The second time the odd number task was played during VR (e.g., passive VR) and the third time, it was played in VR again (e.g., interactive embodied avatar cyberhands VR). The VR treatment order used for the Phase 2 divided attention task was the opposite order as the VR treatment order used in Phase 1 (thermal pain stimuli), and VR treatment order was thus also randomized.

#### Phase 2

To summarize, the Odd Number Task is a divided attention task that involves monitoring auditory numbers during No VR (for 2 min) vs. passive VR with no cyberhands (for 2 min) vs. during interactive cyberhands avatar VR (for two minutes). During the “dual task”, participants must perform the two tasks at the same time, being in VR was one task, and monitoring the odd number task was a competing task. In each condition, participants listened to an auditory string of numbers from 1 to 10, and said “now” every time they heard three odd numbers in a row. They were told that the researcher would be monitoring their accuracy on the odd number task. During the odd number task, the researcher had a printout of the number sequence, and the researcher made a mark to indicate every time the participant said “now” in each of the following conditions.

#### No VR + odd number task

Participants did not wear a VR helmet during the No VR condition. They monitored the odd number task with No VR.

#### Passive VR condition + odd number task

During the passive VR condition, participants went into a virtual living room, and were instructed to look at the green chair, coffee table and the books on the coffee table in VR. They could not see any cyberhands or avatar in virtual reality in this treatment condition (passive, no cyberhands). While they were in passive VR during the Phase 2 divided attention task, as they were in VR looking at the living room chair scene, they also listened to an auditory string of numbers, and said “now” any time they heard three odd numbers in a row.

#### Interactive embodied cyberhands avatar condition + odd number task

While they were in VR during the Phase 2 divided attention task, they looked at the living room chair scene, they used their cyberhands to pick up books in VR, and wiggled their cyberfingers at the same time they listened to an auditory string of numbers, and said “now” any time they heard three odd numbers in a row.

### Power analysis

A power analysis to determine the number of participants needed to test our primary hypothesis was computed apriori, using the statistical program GPower 3.10. The following assumptions were used in the power analyses, all determined from pilot data and Wender et al. 2009^41^, an effect size (d) of .78, power of .95, and an alpha of .05. Under these conditions, we would require 24 participants in the main study to be able to detect a significant treatment effect, and to show that interactive cyberhands treatment was more effective than passive VR for reducing acute worst pain during the brief thermal pain stimuli.

## Results

### Pre-analysis for carry-over effects.

When using a within-subjects design, carry over effects sometimes occur when the effect of the first treatment condition persists, and influences the responses of the second treatment, so that the observed difference between the treatment conditions depends on the treatment order^30^.

The following pre-analysis was conducted to see if there were carry over effects (i.e., to see if the difference between passive VR and interactive avatar VR was stronger depending on which treatment order (passive 1st or passive 2nd). As shown below, non-parametric comparisons were used to test if there were undesired carryover treatment order effects. The difference between Passive VR vs. interactive cyberhands was the dependent variable, and treatment order was the between groups factor (people who received passive VR 1st were considered one group, and those who received passive VR second were considered a second group). No significant interaction was found between treatment order and worst pain ratings (i.e., no significant treatment order effects for Worst pain). No significant interaction was found between treatment order and pain unpleasantness. No significant interaction was found between treatment order and participants’ ratings of Time spent thinking about pain during the thermal stimulus. And finally, no significant interaction was found between treatment order and participants ratings of Fun during the thermal stimulus.

**Table:** 

Test statistics^a^				
	worstdif	thinkdif	unpldif	fundif
Mann–Whitney U	54.000	62.500	56.000	46.500
Wilcoxon W	132.000	128.500	134.000	124.500
Z	− .766	− .219	− .646	− 1.234
Asymp. Sig. (2-tailed)	.444	.827	.518	.217
Exact Sig. [2*(1-tailed Sig.)]	.487^b^	.833^b^	.566^b^	.235^b^
^a^Grouping variable: (Passive 1st = Group 1), (Interactive avatar 1st = Group 2) ^b^Not corrected for ties				

**Figure 5:**
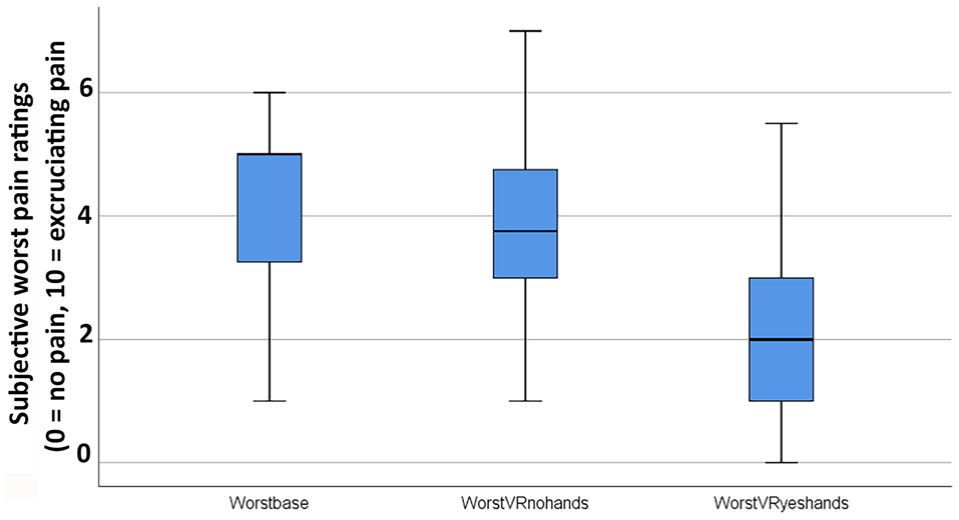
Phase 1. Compared to a passive VR version of the same world, interactive avatar VR was significantly more effective at reducing pain intensity (worst pain ratings).

**Figure 6:**
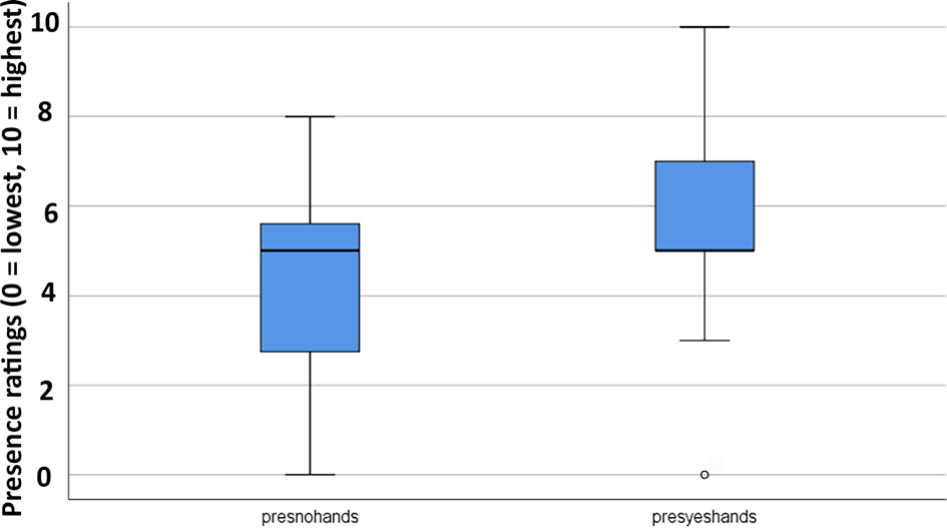
Boxplot showing lower quartile, median, upper quartile, and outliers for Phase 1 results. Interactive avatar VR was significantly more effective at increasing the illusion of “being there” in the virtual world.

**Figure 7:**
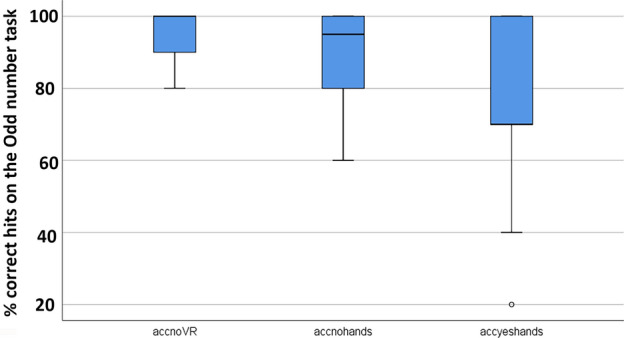
Compared to passive VR, interactive avatar VR significantly reduced participants’ accuracy on an attention demanding task.

Since there were no carry over effects, all of the following analyses were collapsed across treatment order (see Figs. 5, 6 and 7).

#### Worst pain (the primary dependent variable)

A Friedman test showed a significant main effect of No VR vs. passive VR vs. interactive cyberhands VR for the primary dependent measure, Worst Pain, **χ2** (2) = 31.74, *p* = .000 (see Fig. 5). Post-hoc paired comparisons (Wilcoxin signed rank tests) are shown below. (1) Comparing No VR vs. passive VR, worst pain ratings were NOT significantly lower during passive VR, but showed the predicted pattern. (2) Comparing No VR vs interactive cyberhands VR, worst pain was significantly lower during interactive cyberhands VR. And (3) Most importantly, comparing passive VR vs. interactive cyberhands VR, worst pain was significantly lower during interactive cyberhands VR.

Primary dependent variable

Worst pain, mean ratings on a zero to 10 graphic rating scale (SD in parentheses)

**Table:** 

No	VR (baseline)	Passive VR	Interactive cyberhands avatar VR	Wilcoxin signed rank tests
1	4.25 (1.37)	3.60 (1.47)		Z = 1.64, *p* = .10 NS
2	4.25 (1.37)		2.10 (1.48)	Z = 4.04, *p* = .000
3		3.60 (1.47)	2.10 (1.48)	Z = 3.98, *p* = .000

### Pain unpleasantness (a secondary pain measure)

A Friedman test showed a significant main effect of No VR vs. passive VR vs. interactive cyberhands VR for pain unpleasantness, **χ2** (2) = 34.87, *p* = .000.

Post hoc paired comparisons (Wilcoxin signed rank tests) for the variable “pain unpleasantness” are shown below. (1) Comparing No VR vs. passive VR, pain unpleasantness was significantly lower during passive VR. (2) Comparing No VR vs. interactive cyberhands VR, pain unpleasantness was significantly lower during interactive cyberhands VR. And (3) Most importantly, as predicted, comparing passive VR vs. interactive cyberhands VR, pain unpleasantness was significantly lower during interactive cyberhands VR.

Unpleasantness, mean ratings on a zero to 10 graphic rating scale (SD in parentheses).

**Table:** 

No	VR (baseline)	Passive VR	Interactive cyberhands avatar VR	Wilcoxin signed rank tests
1	4.64 (1.20)	3.60 (1.48)		Z = 2.94, *p* = .003
2	4.64 (1.20)		1.94 (1.56)	Z = 4.28, *p* = .000
3		3.60 (1.48)	1.94 (1.56)	Z = 4.04, *p* = .000

### Time spent thinking about pain (a secondary pain measure)

A Friedman Test showed a significant main effect of No VR vs. passive VR vs. interactive cyberhands VR for time spent thinking about pain, **χ2** (2) = 31.17, *p* = .000.

Post hoc paired comparisons (Wilcoxin signed rank tests) for the variable “time spent thinking about pain” are shown below. (1) Although in the predicted direction, No VR vs. passive VR was nonsignificant. (2) Comparing No VR vs. interactive cyberhands VR, time spent thinking about pain was significantly lower during interactive cyberhands VR. And (3) Most importantly, as predicted, comparing passive VR vs. interactive cyberhands VR, time spent thinking about pain was significantly lower during interactive cyberhands VR.

### Time spent thinking about pain

**Table:** 

No	VR (baseline)	Passive VR	Interactive cyberhands avatar VR	Wilcoxin signed rank tests
1	5.03 (1.83)	4.25 (1.62)		Z = 1.36, *p* = .18, NS
2	5.03 (1.83)		1.98 (1.74)	Z = 4.00, *p* = .000
3		4.25(1.62)	1.98 (1.74)	Z = 4.03, *p* = .000

#### Fun

A Friedman Test showed a significant main effect of No VR vs. passive VR vs. interactive cyberhands VR for Fun during the thermal stimulus, **χ2** (2) = 30.61, *p* = .000.

Post hoc paired comparisons (Wilcoxin signed rank tests) for the variable “Fun” are shown below. (1) comparing No VR vs. passive VR, participants reported having significantly more fun during passive VR. (2) Compared to No VR, interactive cyberhands VR was significantly more fun. And (3) Most importantly, compared to passive VR, interactive cyberhands VR was significantly more fun.

Fun during the thermal stimulus, mean ratings on a zero to 10 graphics rating scale (SD in parentheses).

**Table:** 

No	VR (baseline)	Passive VR	Interactive cyberhands avatar VR	Wilcoxin signed rank tests
1	3.31 (2.66)	4.42 (2.59)		Z = − 3.11, *p* = .002
2	3.31 (2.66)		6.05 (2.67)	Z = − 4.01, *p* = .000
3		4.42(2.59)	6.05 (2.67)	Z = − 3.55, *p* = .000

### Presence

Compared to their illusion of presence during passive VR, participants reported having a significantly stronger illusion of presence in virtual reality (being there), during interactive cyberhands VR (where higher presence ratings are better, on a zero to ten rating scale).

Presence in virtual reality

**Table:** 

Passive VR	Interactive cyberhands avatar VR	Wilcoxin Signed Rank Test
4.42 (2.33)	5.76 (2.16)	Z = − 3.32, *p* = .001

How real were the objects in virtual reality

**Table:** 

Passive VR	Interactive cyberhands avatar VR	Wilcoxin Signed Rank Test
4.87 (1.91)	5.48(1.87)	Z = − 2.65, *p* = .008

Nausea in VR

**Table:** 

Passive VR	Interactive cyberhands avatar VR	Wilcoxin Signed Rank Test
0.31 (70)	0.39 (.89)	Z = − .85, *p* = .40 NS

#### Phase 2: performance on the traditional divided attention task

In order to measure participants’ accuracy on the odd number divided attention task, participants listened to a string of auditory numbers and participants said “now” any time they heard three odd numbers in a row.

A Friedman Test showed a significant main effect of No VR vs. passive VR vs. interactive cyberhands VR for accuracy (mean number of hits out of 10 possible hits) on the odd number task, **χ2** (2) = 17.47, *p* = .000.

Accuracy (mean number of hits on the odd number task out of 10 possible hits during each 2 min odd number session (e.g., 9/10 hits = 9.00 = 90% accurate, with SD in parentheses).

**Table:** 

No VR	Passive VR	Interactive Cyberhand VR	Wilcoxin Signed Rank Test
9.60 (.68)	9.00 (1.26)		Z = 2.08, *p* = .038
9.62 (.68)		7.62 (2.13)	Z = 3.39, *p* = .001
	9.05 (1.24)	7.67 (2.13)	Z = 2.69, *p* = .007

### Exploratory side study

To test some important assumptions of our thermal pain paradigm, pilot data collected from 12 new participants from the same subject pool, who were not involved in the main study, received No VR during baseline, passive VR during thermal Test 1 vs. passive VR again during thermal Test 2. As predicted, participants’ pain ratings from the thermal pain stimulations were stable over repeated test pain stimulations for people who received one baseline pain and two test pain stimuli with passive VR, using the same thermal pain paradigm as the main study.

**Figure 8:**
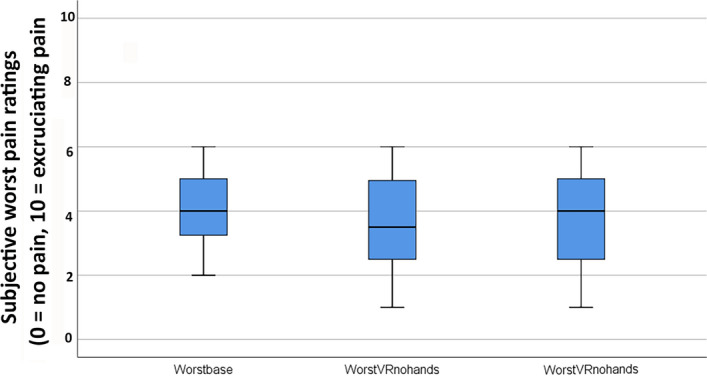
Phase 1. On an n = 12 side study to test important assumptions of my thermal pain paradigm, consistent with my assumptions, as predicted, no significant differences were found between No VR, passive VR on Test 1 vs. passive VR again on Test 2 reducing pain intensity (worst pain ratings).

As shown below, several non-parametric Friedman tests indicated no significant difference between No VR, passive VR on Test 1 vs. passive VR again on Test 2 for worst pain, pain unpleasantness, time spent thinking about pain, or fun during the pain stimulus. In other words, fortunately there was no evidence of habituation to the thermal pain stimulus. For this side pilot study, the same thermal stimulus elicited similar ratings for each of the three pain stimuli (Fig. 8).

**Table:** 

Worst pain during No VR = 3.96 (1.25)
Worst pain during passive VR 1st time = 3.58 (1.50)
Worst pain during passive VR 2nd time = 3.72 (1.48)
**χ2 (2) = .65, *p* = .72 NS**
Pain Unpleasantness during No VR = 3.97 (1.13)
Pain Unpleasantness during passive VR 1st time = 3.67 (1.44)
Pain Unpleasantness during passive VR 2nd time = 3.63 (1.55)
**χ2 (2) = 1.90, *p* = .39 NS**
Time spent thinking about pain during No VR = 4.75 (2.26)
Time spent thinking about pain during passive VR 1st time = 3.79 (2.46)
Time spent thinking about pain during passive VR 2nd time = 3.88 (1.84)
**χ2 (2) = 2.23, *p* = .33 NS**
Fun during pain stimulus during No VR = 3.83 (2.52)
Fun during pain stimulus during passive VR 1st time = 4.38 (2.69)
Fun during pain stimulus during passive VR 2nd time = 3.96 (2.60)
**χ2 (2) = 1.81, *p* = .41 NS**

## Discussion

In the current study, as predicted, interacting with virtual objects via embodied avatar hands (i.e., avatar VR) significantly increased the participant’s illusion of “being there” in the virtual world, increased VR analgesia, and increased fun during the pain stimulus. The current study also measured for the first time, how much attention was diverted by avatar VR. Consistent with the notion that interactive embodied immersive VR is unusually attention grabbing, during Phase 2, participants made significantly more errors on the divided attention task during the interactive avatar VR condition, compared to passive VR. These results implicate an attentional mechanism for how VR reduces pain, and help understand how VR influences pain perception.

Although immersive virtual reality can often help reduce the acute pain of patients during painful medical procedures, some medical procedures are so painful that extra strength VR analgesia may be needed^46^. The current study measured whether a stronger (more immersive) dose of virtual reality could increase analgesia. Our results indicate that the immersiveness of a VR system can be increased substantially, (e.g., via avatars) with little or no increase in VR side effects, unlike opioids, which show a dose–response increase in side effects (e.g., increased nausea and constipation) with higher doses, and opioid side effects linger for hours after the medical procedure. Immersive interactive cyberhand avatar embodiment with extra wide field of view VR helmet (180 degrees field of view, high resolution) appears to have considerable potential as a short acting extra strength VR analgesia system. A number of studies have now shown that increasing the immersiveness of the VR system increases VR analgesia for acute^38–41^. In addition, the relationship between having a body/avatar in VR and presence has been shown previously^34,47^ and the current results contribute to those findings.

However, according to Trost et al. 2020^26^, there is currently a gap in the scientific literature on the use of immersive VR avatars to treat acute pain. To date, nearly all studies involving virtual embodiment have targeted treatments designed for chronic pain patients, (e.g., phantom limb pain)^26,48–54^. The current study is one of the first VR studies designed to treat acute pain that includes an avatar^14,16,55^, and is the first acute pain VR analgesia study to manipulate embodiment, and to measure whether interactive embodiment with cyberhands avatars increases analgesia (and reduces accuracy on the odd number task, an objective measure of attention), compared to passive VR with no avatar, a promising direction for future research.

The current study has a number of limitations that should be taken into consideration when interpreting the results. The within-subjects design is statistically powerful and allows each participant to compare the different treatments, but has the drawback that participants remain aware of the different treatment conditions, and this awareness has the potential to influence the results^56^. The current study should thus be replicated using a between groups design, ideally with participants blinded to VR treatment group conditions^57^, and using a larger sample size.

Even though the sense of presence in the VR environment was assessed among the participants, it would be interesting (in future investigations) to also measure how strong the sense of embodiment was in terms of body ownership, agency, and self-location to investigate possible relationships with the distraction from pain effect. Another limitation is that the current study is an analog laboratory pain study, with only brief thermal pain stimuli and brief VR treatment durations. Whether the current results generalize to clinical settings (e.g., severely burned children during 20 min burn wound debridement sessions) is an important research topic for future clinical studies with patients during painful medical procedures, e.g.,^2,58^.

Despite these limitations, the results of this study could also have other important clinical implications. According to Keefe et al.^1^, the epidemic of opioid related overdose deaths^7,59^ has greatly increased the urgency to develop effective non-drug pain control techniques that can help reduce the medical community’s current heavy reliance on opioid analgesics for pain control. Although to date VR analgesia has typically been used adjunctively in addition to traditional pain medications, a stronger version of VR has the potential to reduce the use of opioids^46,60,61^ and/or to help compensate for increasing tendencies to under-medicate patients, as opioids become much more strictly controlled, due to recent increases in federal regulation of opioid prescription.

Future Directions. According to Matamala-Gomez et al, p 1^62^ “Using such virtual embodiment to manipulate body perception is starting to be extensively investigated and may have clinical implications for conditions that involve altered body image such as chronic pain.” Researchers are currently developing/exploring the use of high-tech immersive virtual reality embodiment therapy (e.g., embodied avatar cyberhands) for phantom limb pain and other chronic pain diseases, to create a more compelling illusion of limb ownership and to help treat phantom limb pain more effectively. There is some evidence that for some chronic pain patients, illusions can be used to help reduce persistent pain, and perhaps normalize maladaptive pathological distortions of the patient’s homunculus^51,62^.

Patients wearing a VR helmet look down and see two virtual arms and two virtual hands. They are able to use their virtual body/hand to interact with objects in the virtual world. This can potentially be used for both amputees and nonamputee chronic pain patients.

Major computer companies’ multibillion dollar investments into virtual reality technology are making VR goggles widely available and affordable for medical applications, and the VR technologies needed to include avatars into a virtual environment have become much simpler and inexpensive. Avatar technology and the ability to socialize in multi-participant networked VR is rapidly improving.  Additional research and development of VR analgesia is recommended.

## References

[1] Keefe FJ (2012). Virtual reality for persistent pain: a new direction for behavioral pain management. Pain.

[2] Hoffman HG (2019). Immersive virtual reality as an adjunctive non-opioid analgesic for pre-dominantly Latin American children with large severe burn wounds during burn wound cleaning in the intensive care unit: A pilot study. Front. Hum. Neurosci..

[3] Melzack R (1990). The tragedy of needless pain. Sci. Am..

[4] Krane EJ, Walco GA (2019). With apologies to Lennon and McCartney, all we need is data: Opioid concerns in pediatrics. Clin. J. Pain.

[5] Malchow RJ, Black IH (2008). The evolution of pain management in the critically ill trauma patient: Emerging concepts from the global war on terrorism. Crit. Care Med..

[6] McIntyre MK, Clifford JL, Maani CV, Burmeister DM (2016). Progress of clinical practice on the management of burn-associated pain: Lessons from animal models. Burns.

[7] Wilson N, Kariisa M, Seth P, Smith HT, Davis NL (2020). Drug and opioid-involved overdose deaths—United States, 2017–2018. MMWR Morb. Mortal Wkly. Rep..

[8] Birnie KA, Chambers CT, Spellman CM (2017). Mechanisms of distraction in acute pain perception and modulation. Pain.

[9] Donnelly TJ, Palermo TM, Newton-John TRO (2020). Parent cognitive, behavioural, and affective factors and their relation to child pain and functioning in pediatric chronic pain: A systematic review and meta-analysis. Pain.

[10] Fields HL (2018). How expectations influence pain. Pain.

[11] Melzack R, Wall PD (1965). Pain mechanisms: A new theory. Science.

[12] Noel M, Rabbitts JA, Tai GG, Palermo TM (2015). Remembering pain after surgery: A longitudinal examination of the role of pain catastrophizing in children's and parents' recall. Pain.

[13] Topham L (2020). The transition from acute to chronic pain: dynamic epigenetic reprogramming of the mouse prefrontal cortex up to 1 year after nerve injury. Pain.

[14] Hoffman HG, Patterson DR, Carrougher GJ (2000). Use of virtual reality for adjunctive treatment of adult burn pain during physical therapy: A controlled study. Clin J Pain.

[15] Hoffman HG (1998). Virtual reality: a new tool for interdisciplinary psychology research. CyberPsychol. Behav..

[16] Hoffman HG, Doctor JN, Patterson DR, Carrougher GJ, Furness TA (2000). Virtual reality as an adjunctive pain control during burn wound care in adolescent patients. Pain.

[17] Dahlquist LM (2007). Active and passive distraction using a head-mounted display helmet: Effects on cold pressor pain in children. Health Psychol..

[18] Garrett B (2014). A rapid evidence assessment of immersive virtual reality as an adjunct therapy in acute pain management in clinical practice. Clin. J. Pain.

[19] Hoffman HG (2004). Virtual-reality therapy. Sci. Am..

[20] Jeffs D (2014). Effect of virtual reality on adolescent pain during burn wound care. J. Burn Care Res..

[21] Kathner I, Bader T, Pauli P (2019). Heat pain modulation with virtual water during a virtual hand illusion. Sci. Rep..

[22] Khadra C (2020). Effects of a projector-based hybrid virtual reality on pain in young children with burn injuries during hydrotherapy sessions: A within-subject randomized crossover trial. Burns.

[23] Maani CV (2011). Virtual reality pain control during burn wound debridement of combat-related burn injuries using robot-like arm mounted VR goggles. J. Trauma.

[24] Maani CV (2011). Combining ketamine and virtual reality pain control during severe burn wound care: One military and one civilian patient. Pain Med..

[25] Lier EJ, Oosterman JM, Assmann R, de Vries M, van Goor H (2020). The effect of Virtual Reality on evoked potentials following painful electrical stimuli and subjective pain. Sci. Rep..

[26] Trost Z, France C, Anam M, Shum C (2021). Virtual reality approaches to pain: Toward a state of the science. Pain.

[27] Hoffman HG (2004). Modulation of thermal pain-related brain activity with virtual reality: Evidence from fMRI. NeuroReport.

[28] Hoffman HG (2007). The analgesic effects of opioids and immersive virtual reality distraction: evidence from subjective and functional brain imaging assessments. Anesth Analg.

[29] Bergstrom I, Kilteni K, Slater M (2016). First-person perspective virtual body posture influences stress: A virtual reality body ownership study. PLoS ONE.

[30] Dwan K, Li T, Altman DG, Elbourne D (2019). CONSORT 2010 statement: extension to randomised crossover trials. BMJ.

[31] Hoffman HG (2020). Virtual reality hand therapy: A new tool for nonopioid analgesia for acute procedural pain, hand rehabilitation, and VR embodiment therapy for phantom limb pain. J. Hand Ther..

[32] Jensen MP (2003). The validity and reliability of pain measures in adults with cancer. J. Pain.

[33] Williamson A, Hoggart B (2005). Pain: a review of three commonly used pain rating scales. J. Clin. Nurs..

[34] Slater M, Spanlang B, Corominas D (2010). Simulating virtual environments within virtual environments as the basis for a psychophysics of presence. ACM Trans. Graphic.

[35] Slater M, Usoh M, Steed A (1994). Depth of presence in immersive virtual environments. Presence Teleoper. Virtual Environ..

[36] Slater M, Wilbur S (1997). A framework for immersive virtual environments (FIVE): speculations on the role of presence in virtual environments. Presence Teleoper. Virtual Environ..

[37] Hoffman HG (2006). Using FMRI to study the neural correlates of virtual reality analgesia. CNS Spectr..

[38] Al-Ghamdi NA (2019). Virtual reality analgesia with interactive eye tracking during brief thermal pain stimuli: A randomized controlled trial (crossover design). Front. Hum. Neurosci..

[39] Hoffman HG (2004). Manipulating presence influences the magnitude of virtual reality analgesia. Pain.

[40] Hoffman HG (2006). Virtual reality helmet display quality influences the magnitude of virtual reality analgesia. J. Pain.

[41] Wender R (2009). Interactivity influences the magnitude of virtual reality analgesia. J. Cyber Ther. Rehabil..

[42] Craik F (1983). On the transfer of information from temporary to permanent memory. Philos. Trans. R. Soc. B.

[43] Hoffman HG, Garcia-Palacios A, Kapa V, Beecher J, Sharar SR (2003). Immersive virtual reality for reducing experimental ischemic pain. Int. J. Hum. Comput. Int..

[44] Jacoby LL, Woloshyn V, Kelley C (1989). Becoming famous without being recognized—Unconscious influences of memory produced by dividing attention. J. Exp. Psychol. Gen..

[45] Iidaka T, Anderson ND, Kapur S, Cabeza R, Craik FI (2000). The effect of divided attention on encoding and retrieval in episodic memory revealed by positron emission tomography. J. Cogn. Neurosci..

[46] Firoozabadi R (2020). Case report: Virtual reality analgesia in an opioid sparing orthopedic outpatient clinic setting: A case study. Front. Virtual Real.

[47] Slater M, Usoh M (1993). Representations Systems, Perceptual Position, and Presence in Immersive Virtual Environments. Presence Teleoper. Virtual Environ..

[48] Martini M (2016). Real, rubber or virtual: The vision of "one's own" body as a means for pain modulation. A narrative review. Conscious Cogn..

[49] Martini M, Kilteni K, Maselli A, Sanchez-Vives MV (2015). The body fades away: investigating the effects of transparency of an embodied virtual body on pain threshold and body ownership. Sci. Rep..

[50] Martini M, Perez-Marcos D, Sanchez-Vives MV (2013). What color is my arm? Changes in skin color of an embodied virtual arm modulates pain threshold. Front. Hum. Neurosci..

[51] Matamala-Gomez M, DiazGonzalez AM, Slater M, Sanchez-Vives MV (2019). Decreasing pain ratings in chronic arm pain through changing a virtual body. Different strategies for different pain types. J. Pain.

[52] Matamala-Gomez M (2020). Changing body representation through full body ownership illusions might foster motor rehabilitation outcome in patients with stroke. Front. Psychol..

[53] Matamala-Gomez M, Nierula B, Donegan T, Slater M, Sanchez-Vives MV (2020). Manipulating the perceived shape and color of a virtual limb can modulate pain responses. J. Clin. Med..

[54] Solca M (2018). Heartbeat-enhanced immersive virtual reality to treat complex regional pain syndrome. Neurology.

[55] Hoffman HG, Patterson DR, Carrougher GJ, Sharar SR (2001). Effectiveness of virtual reality-based pain control with multiple treatments. Clin. J. Pain.

[56] Campbell D, Stanley JC (1963). Experimental and Quasi-Experimental Designs for Research.

[57] Schulz KF, Grimes DA (2002). Blinding in randomised trials: hiding who got what. Lancet.

[58] Hoffman HG (2020). Virtual reality analgesia for children with large severe burn wounds during burn wound debridement. Front. Virtual Real..

[59] Ballantyne JC (2018). The brain on opioids. Pain.

[60] Kipping B, Rodger S, Miller K, Kimble RM (2012). Virtual reality for acute pain reduction in adolescents undergoing burn wound care: A prospective randomized controlled trial. Burns.

[61] McSherry T (2018). Randomized, crossover study of immersive virtual reality to decrease opioid use during painful wound care procedures in adults. J Burn Care Res.

[62] Matamala-Gomez M (2019). Immersive virtual reality and virtual embodiment for pain relief. Front. Hum. Neurosci..

